# Impact of Different Nanoparticles on Common Wheat (*Triticum aestivum* L.) Plants, Course, and Intensity of Photosynthesis

**DOI:** 10.1155/2022/3693869

**Published:** 2022-11-09

**Authors:** Lauris Jankovskis, Inese Kokina, Ilona Plaksenkova, Marija Jermaļonoka

**Affiliations:** Department of Biotechnology, Laboratory of Genomics and Biotechnology, Daugavpils University, Parādes str. 1A, 5401-Daugavpils, Latvia

## Abstract

The size of nanoparticles (NPs) allows them to accumulate in plants, and they affect plant growth by altering the size of leaves and roots and affecting their photosynthetic reactions by altering the composition of proteins in the electron transport chain, chlorophyll biosynthesis, and carbohydrate synthesis reactions. Plants play an important role on Earth as nutrient producers in all trophic food webs by producing oxygen, absorbing carbon dioxide, and synthesizing edible carbohydrates during photosynthesis. In this study, Fe_3_O_4_ and ZnO NPs at various concentrations (0, 1, 2, and 4 mg/l) were used to investigate the effect of NPs on plant morphological parameters and photosynthesis intensity, determining the amount of chlorophyll and the absorption of their light spectrum in common wheat (*Triticum aestivum* L.). Fe_3_O_4_ (25 nm, 2 mg/l, and 4 mg/l) and ZnO (32 nm, 4 mg/l) significantly increased the leaf length of common wheat seedlings. However, Fe_3_O_4_ NPs (25 nm, 1 mg/l, and 4 mg/l) significantly reduced light absorption at 645 and 663 nm and the content of chlorophyll b, chlorophyll a, and total chlorophyll, but Fe_3_O_4_ (25 nm, 2 mg/l) significantly reduced the chlorophyll a content. In addition, ZnO NPs (32 nm, 1 mg/l) significantly increased the chlorophyll b content. This study has made a major contribution to understanding the effect of low concentrations of NPs on plant seedlings. Currently, NPs with high concentrations, starting at 10 mg/l, have been analysed in other studies, but in the environment, NPs enter plants in low concentrations as dust or through water droplets. Therefore, it is important to determine the potential impact of small concentrations of NPs on crops that are important for agriculture.

## 1. Introduction

Nanoparticles (NPs) are insoluble, persistent, and inseparable nanomaterials with a size of 1 to 100 nm [1, 2]. NPs are common in the environment in various concentrations, sizes, and shapes [3, 4]. Although NP research began only in the 20th century [5], there is now a broad classification of NPs, such as organic and inorganic or natural and artificially synthesised [6]. NPs of oxides, especially Fe_3_O_4_, ZnO, and SiO_2_, are currently relevant in research [7–10]. Due to the intensive production of electronic equipment and technology and the results of oxidation processes, NPs enter the environment from industrial waste [11].

NPs enter plants by roots, leaves, or stems through the cuticle, epidermis, cytoplasm, intercellular space, along and around cell walls, or through the plasma membrane or vascular cells [12]. NP transport takes place together with water through the vascular tissues of the plant xylem or the free intercellular space and cell membranes in the ascending direction. Through phloem filaments, NPs can move in all directions, allowing them to penetrate all parts of the plant [13]. NPs localise in all cellular structures, mostly in the roots, but some NPs can migrate to other parts of the plant [14].

Currently, research on the occurrence of NPs, their penetration into living organisms, and their impact on physiological processes, especially plant photosynthesis, which produces oxygen, edible carbohydrates, and reduces CO_2_, is relevant [15].

Research has shown that NPs can affect plant morphological parameters and the intensity of photosynthesis [16]. Positive or negative effects are determined by the size, concentration, and starting material of the NPs [17]. Low or medium concentrations of Fe_3_O_4_, ZnO, and SiO_2_ NPs promote plant growth and development, accelerate oxygen production, and intensify CO_2_ absorption. Low concentrations of ZnO and iron oxide NPs (20 mg/l) provide Zn_2_^+^microelements and iron ions for development, flowering, and leaf and root growth [18]. Fe_3_O_4_ improves seed germination, increases chlorophyll content, promotes the photochemical efficiency (Fv/Fm) of PSII, and increases the amount of chlorophyll a and b, as well as promoting the expression of genes for enzymes involved in photosynthesis [19]. ZnO NPs inhibit the growth of microorganisms (bacteria and fungi) by penetrating their cell membranes. Oxidative stress damages bacterial lipids, carbohydrates, proteins, and DNA; thus, ZnO is used in plant protection products [20].

However, if any of the concentrations of oxide NPs are high (>50 mg/l), then cytotoxicity and genotoxicity develop and damage various cells [21]. For example, ZnO-induced lipid peroxidation increases the expression of a gene that encodes a tonoplast protein. The tonoplast surrounds the vacuole and allows substances to pass through it. If the ZnO content increases, the transport of substances in the vacuole decreases, and Zn compounds may accumulate more in the vacuole. Impaired vacuoles lead to decreased photosynthetic intensity, decreased transpiration, and decreased water conductivity [12] Fe_3_O_4_ NPs can also oxidise at high concentrations above 50 mg/l and significantly reduce the fresh and dry weight of roots and leaves, as well as reduce the content of carotenoids and chlorophyll a and b in leaves, promoting chloroplast cell degradation [22].

## 2. Materials and Methods

### 2.1. Chemical

Fe_3_O_4_ NPs with a diameter of 25 nm were synthesised at G. Liberta's Innovative Microscopy Center using an aqueous ammonium hydroxide solution and ferric chloride (II) and (III) in a ratio of 1 : 2 by the coprecipitation (Massart) method. In this experiment, 0.0429 g of FeCl_2_ × 4H_2_O and 0.167 g of FeCl_3_ × 6H_2_O were dissolved in 50 mL of distilled water. Dropwise and with constant stirring, 0.27 mL of 25% NH_4_OH was added to the solution. An aqueous citric acid solution (40 mg/mL, 2 mL) was used to stabilize nanostructures. A permanent magnet was used for the separation of the precipitate. To remove residual reagents, the precipitate was rinsed three times with distilled water. The production of Fe_3_O_4_ NPs can be described schematically by the following equation [23]:

(1) $$\begin{array}{c} {Fe}^{2+}+2{Fe}^{3+}+8{OH}^{-}={Fe}_{3}O_{4}↓4H_{2}O. \end{array}$$

ZnO NPs with a diameter of 32 nm were synthesised by preparing 2 stock solutions. At first, 0.1 M Zn (CH_3_COO)2 × 2H_2_O (Sigma-Aldrich, ≥98%) was dissolved with continuous stirring in 50 mL of ethanol. Then, 25 mL of 0.2 M NaOH (Merck, ≥99%) was also dissolved in ethanol and then dropwise added to the first stock solution until the obtained solution reached a pH value of 11. The solution was ultrasonically stirred for one hour before being poured into a sealed Teflon-lined beaker. Solution heating in an oven preheated to 90°C for six hours produced a white precipitate, which was rinsed with distilled water and dried in the oven at 90°C. The white powder, with NP agglomerates, was diluted in water to the following concentrations: 1, 2, and 4 mg/L [24].

### 2.2. Common Wheat Seedling Cultivation

**Figure 1:**
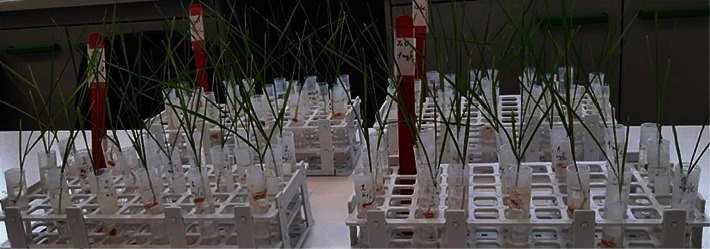
Samples of common wheat (*Triticum aestivum* L.) seedlings grown in hydroponics for 15 days with different concentrations: 1, 2, and 4 mg/l of ZnO nanoparticles.

Soft spring-crop wheat “Jasna” seeds were purchased from the Institute of Agricultural Resources and Economics, Stende Research Center (Priekuli, Latvia). Seeds were germinated in water only on filter paper to exclude the effect of other nutrients on the growth of the seedlings. After a period of germination and growth of about 8 days, they were placed in a growth chamber at +24°C and grown in hydroponics in the experimental groups, ZnO or Fe_3_O_4_, hydroponically for 15 days. The control group was grown without NPs. Forty seedlings were grown in each plant group, as shown in Figure 1.

### 2.3. Morphological Analysis of Common Wheat Seedlings

Seedling morphological parameters were determined for all seedling groups used in the experiment. The samples selected from each group were free from lesions and pathogens. All measurements were obtained for seedlings grown hydroponically with NPs for 15 days. The length of the leaves of the shoots and roots was measured using a ruler. The number of root shoots was also counted.

### 2.4. Preparation of Plant Suspension for Chlorophyll Extraction

Seedlings were cut into fine pieces, and 100 mg of common wheat green leaves were weighed for each wheat seedling; 5 ml of 96.6% ethanol was added to the weighed leaves. The mixture was then ground with a pestle. Using a new filter for each sample, the resulting mixture was filtered. Before centrifugation, the mixture was kept in the dark for 30 minutes.

### 2.5. Determination of Photosynthesis Intensity by Spectrophotometry

The absorbance of the chlorophyll in the chlorophyll solution was determined using a NanoDrop 1000 spectrophotometer (Thermo Scientific, Wilmington, North Carolina, USA). Three replicates of the measurements were performed for each sample. The obtained data were processed using the ND-1000 V3.6.0 computer programme at light wavelengths of 645 and 663 nm for chlorophyll b and a, respectively [19].

The chlorophyll content was calculated for each measurement using the following equations:

(2) $$\begin{array}{c} total\;chlorophyll=\frac{\left(\left(20.2\times A645\right)+\left(8.02\times A663\right)\right)}{1000\times W\times a}\times V, \end{array}$$

(3) $$\begin{array}{c} chlorophyll\;a=\frac{\left(\left(12.7\times A663\right)-\left(2.69\times A645\right)\right)}{1000\times W\times a}\times V, \end{array}$$

(4) $$\begin{array}{c} chlorophyll\;b=\frac{\left(\left(22.9\times A645\right)-\left(4.86\times A663\right)\right)}{1000\times W\times a}\times V\text{,} \end{array}$$

where *A* is the absorption in a spectrophotometer at a specific wavelength (nm); *W* is the mass of the fresh sample (g); *V* is the volume of the centrifuged suspension (ml); *α* is the light path length in the chlorophyll cell. The unit of chlorophyll in the SI system is mg/g [19].

### 2.6. Statistical Analysis

The mean, standard deviation (SD), and one-way analysis of variance (ANOVA) were performed to determine the differences and significance (*P* < 0.05) of morphological measurements and total chlorophyll and chlorophyll b and a data.

## 3. Results

### 3.1. Morphological Analysis of Common Wheat Seedlings after Exposure to Fe_3_O_4_ and ZnO NPs

The effect of three different concentrations (1, 2, and 4 mg/l) of Fe_3_O_4_ and ZnO NPs on the length of wheat leaves and roots, as well as the number of root shoots, was investigated.

**Figure 2:**
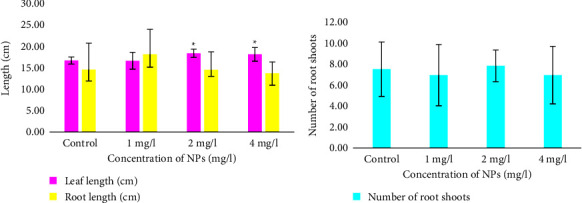
Leaf and root lengths (a) and number of roots (b) in common wheat (*Triticum aestivum* L.) after 15 days of treatment with Fe_3_O_4_ nanoparticles. The values are the means of three replicates with a standard deviation. ^∗^indicates a statistically significant difference compared to the control (*P* < 0.05).

**Figure 3:**
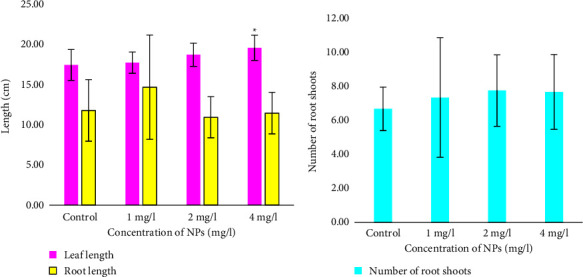
Leaf and root lengths (a) and a number of roots (b) of common wheat (*Triticum aestivum* L.) after 15 days of treatment with ZnO NPs. The values are the means of three replicates with a standard deviation. ^∗^indicates a statistically significant difference compared to the control (*P* < 0.05).

**Figure 4:**
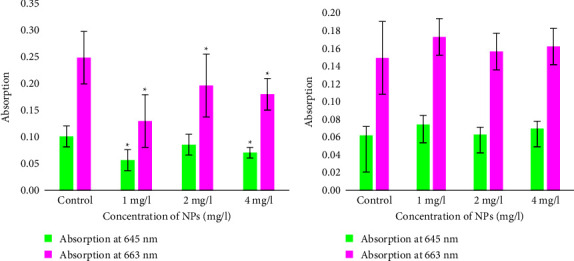
Absorption of common wheat (*Triticum aestivum* L.) at 645 nm (for chlorophyll b) and 663 nm (for chlorophyll a) 15 days after treatment with Fe3O4 (a) and ZnO (b) nanoparticles. The values are the mean of three replicates with the standard deviation. ^∗^indicates a statistically significant difference (*P*<0.05) compared to the control.

The average leaf length for seedlings with 2 and 4 mg/l Fe_3_O_4_ NPs (Figure 2(a)) and 4 mg/l ZnO NPs (Figure 3(a)) was significantly increased in comparison with the control samples. A concentration of 2 mg/l of Fe_3_O_4_ NPs was the minimum at which a significant effect of Fe_3_O_4_ was observed. In contrast to ZnO NPs, the minimum concentration at which a significant effect was observed was 4 mg/l. Wheat plants with the maximum available concentration of ZnO NPs showed the longest average leaf length of 19.7 ± 1.58 cm, which was 2.1 cm more than the control plants.

Additionally, long leaves were observed in seedlings growing with 2 mg/l (18.6 ± 0.85 cm) and 4 mg/l Fe_3_O_4_ NPs (18.4 ± 1.60 cm). However, the average results for plants with 2 and 4 mg/l Fe_3_O_4_ NPs were very similar.

In contrast, leaf lengths for seedlings with 1 mg/l Fe_3_O_4_ NPs and seedlings with 1 and 2 mg/l ZnO NPs (Figure 3(a)) were not significantly different from the control samples. Plants treated with 1 mg/l ZnO NPs showed small leaf extensions of 0.3 cm, and at 2 mg/l ZnO NPs, they showed an increase of 1.2 cm. The leaf length of the seedlings treated with Fe_3_O_4_ NPs at a concentration of 1 mg/l and the control seedlings (Figure 2(a)) was 16.9 ± 1.98 cm.

The average root lengths of the plants with Fe_3_O_4_ NPs (Figure 2(a)) and ZnO NPs (Figure 3(a)) at 1 mg/l had a statistically insignificant (*P* > 0.05) increase in comparison with the control plants. However, the increase was small: 3.6 cm for plants with Fe_3_O_4_ NPs and 2.9 cm for plants with ZnO NPs. At 2 and 4 mg/l, both NPs insignificantly reduced root length compared to the control group.

Similarly, the number of roots (Figure 2(b)) at all Fe_3_O_4_ NPs and ZnO (Figure 3(b)) concentrations did not differ significantly from the control plants. The control plants showed an average number of 7.8 ± 2.67, but plants with Fe_3_O_4_ NPs (2 mg/l) showed an insignificant increase of 0.3. The average number of shoots was the same in plants at 1 and 4 mg/l. These groups insignificantly reduced the number of root shoots by 0.6. Increasing the concentration of NPs did not always decrease the number of shoots.

The control plants showed an average number of shoots of 6.8 ± 1.29. Plants with ZnO NPs (2 mg/l) showed an insignificant increase of 0.6. The average number of shoots was the same in plants at 1 and 4 mg/l. These groups also insignificantly increased the number of root shoots by 1 Increasing the concentration of NPs did not significantly increase the number of shoots.

#### 3.1.1. Determination of Photosynthesis Intensity by Spectrophotometry

*(1) Effect of Fe*. _*3*_*O*_*4*_*and ZnO NPs on the Light Absorption of a Suspension of Common Wheat Seedlings.*The effect of three different concentrations (1, 2, and 4 mg/l) of Fe_3_O_4_ and ZnO NPs on the light absorption of chlorophyll at light wavelengths of 645 and 663 nm (Figure 4) was investigated. Fe_3_O_4_ NPs significantly affected absorption at 645 and 663 nm at almost all available NP concentrations. Fe_3_O_4_ NPs statistically significantly reduced light absorption (*P<*0.05) at 645 nm (for chlorophyll (b) at concentrations of 1 and 4 mg/l.

For the control plants, the mean absorbance at 645 nm was 0.10 ± 0.02. Plants with a concentration of 1 mg/l NPs showed a significant decrease in absorption of 0.06. Plants with 4 mg/l NPs showed a significant decrease in absorbance at 645 nm (0.4). In contrast, plants with 2 mg/l NPs showed a statistically insignificant decrease of 0.01.

Fe_3_O_4_ NPs (Figure 4(a)) significantly reduced light absorption (*P*<0.05) at 663 nm in plants at all available concentrations. Plants with 1 mg/l NPs decreased absorption by half (0.12). Plants with 2 mg/l NPs also showed a significant decrease of 0.05. Plants with 4 mg/l NPs also showed a statistically significant decrease of 0.07 in absorbance at 645 nm compared to the control.

ZnO NPs (Figure 4(b)) did not significantly affect (*P*>0.05) the absorption at both 645 and 663 nm at all available NP concentrations plants with 1 and 4 mg/l NPs showed a very small decrease in absorption at 645 nm (0.01). Plants grown with 2 mg/l NPs showed the same absorption as the control plants (0.06).

ZnO NPs did not significantly affect light absorption at 663 nm (*P*>0.05) at all available NP concentrations. Plants with a concentration of 1 mg/l NPs showed a very low increase in absorption of 0.02. Plants grown with 2 mg/l NPs showed the same absorption as the control plants. Plants with an NP concentration of 4 mg/l also showed a small increase in the absorption of 0.01.

*(2) Chlorophyll a, Chlorophyll b, and Total Chlorophyll Content of Samples of Common Wheat after Exposure to Fe*. _*3*_*O*_*4*_*NPs.* The effect of three different concentrations (1, 2, and 4 mg/l) of Fe_3_O_4_ NPs on chlorophyll a, chlorophyll b, and total chlorophyll was investigated. Fe_3_O_4_ NPs at concentrations of 1 and 4 mg/l significantly reduced the amount of both chlorophyll a and chlorophyll b, and naturally, at these concentrations, the total amount of chlorophyll was reduced in all samples. The largest reduction in chlorophyll was observed in plants with 1 mg/l NPs, where the samples showed a total chlorophyll content of 0.67 ± 0.23 *μ*g/g, which differed from the control by 0.56 *μ*g/g, almost twice the difference. Samples of this concentration naturally had low levels of chlorophyll a and chlorophyll b. The largest difference was seen in the amount of chlorophyll a. Control samples had a chlorophyll a content of 0.88 ± 0.18 and samples treated with 1 mg/l had less than 0.42 *μ*g/g of chlorophyll a. Chlorophyll b decreased by 0.09 *μ*g/g at this concentration. In the samples with 4 mg/l NPs, the amount of chlorophyll a also decreased the most, by 0.24 *μ*g/g compared to the control. The average amount of chlorophyll b in the experimental group was also lower than in the control group, showing a difference of 0.017 *μ*g/g. The mean total chlorophyll amount at this concentration differed significantly from the control mean by 0.35 *μ*g/g, as shown in Table 1.

In samples with a concentration of 2 mg/l NPs, only chlorophyll a was significantly reduced compared to the control (0.88 ± 0.18 and 0.19 *μ*g/g, respectively). However, at this concentration, an insignificant reduction of chlorophyll b by 0.03 *μ*g/g was observed. Thus, at this concentration, the total amount of chlorophyll decreased insignificantly, distinguishing this group from the control group by only 0.22 *μ*g/g. Overall, a negative effect of Fe_3_O_4_ was observed on the amount of chlorophyll in the seedlings.

*(3) Chlorophyll a, Chlorophyll b, and Total Chlorophyll Content of Samples of Common Wheat after Exposure to ZnO NPs*. The effect of three different concentrations (1, 2, and 4 mg/l) of ZnO NPs on chlorophyll a, chlorophyll b, and total chlorophyll was investigated. All concentrations of ZnO NPs increased the chlorophyll a, chlorophyll b, and total chlorophyll content. However, this increase was insignificant in almost all indicators (*P*>0.05). Only the average amount of chlorophyll b in plants with 1 mg/l ZnO NPs differed significantly. The amount of chlorophyll b in the control plants was 0.21 ± 0.065 *μ*g/g, and for the experimental group, it was 0.26 ± 0.051 *μ*g/g. The chlorophyll a content increased by 0.04 *μ*g/g. However, at 1 mg/l, the largest increases in chlorophyll a and total chlorophyll were observed. The chlorophyll a content increased by 0.07 *μ*g/g. The total chlorophyll increased by 0.13 *μ*g/g. However, this increase was statistically insignificant (Table 2).

For plants treated with 2 mg/l ZnO NPs, the content of chlorophyll a and total chlorophyll differed by 0.02 *μ*g/g. The amount of chlorophyll b was equal to that reported in the control plants (0.21 *μ*g/g).

At 4 mg/l ZnO NPs, the chlorophyll a content differed from the control by 0.04 *μ*g/g; the chlorophyll b content also differed very little from the control by only 0.03. The total chlorophyll content was 0.79 ± 0.082 *μ*g/g, which differed insignificantly from the control by 0.08 *μ*g/g.

## 4. Discussion

NPs appeared in nature with the emergence of the universe. Nanotechnology was discovered as a science in the mid-20th century, but intensive NP research did not begin until the 21st century. Over the last 20 years, NPs have become a topical research object in modern science and one of the most frequently discussed topics related to environmental protection [5].

NPs are now known to be used in the manufacture of sensors for various household items and appliances, such as solar cells [25]. They can even be used in medicine for magnetic resonance imaging (MRI) or in the transportation of medicinal products to the human body [17]. The current problem is the uncontrolled presence of nanoparticles in the environment, which naturally occur from materials such as iron ore and sand but are also released into the environment by human activity [3]. It is important to control the synthesis of NPs and the use of existing materials so as not to cause further NP contamination. In the production of new equipment, the use of previously synthesised NPs using green synthesis methods, which involve the secondary extraction of NPs from plants or materials that already contain nanomaterials or NPs, is recommended [26].

Unfortunately, research has also shown that there is a high level of contamination of nanomaterials and NPs in nature, with NPs in nature being in a variety of sizes and shapes. They readily enter both animal and plant cells [13]. When NPs enter plants, their physiological processes are affected. Currently, research on the effect of NPs on the process of photosynthesis is very important. Although indirect studies of the process of photosynthesis began in the 18th century with the observation of the release of air bubbles from aquatic plants, photosynthetic reactions were explained and the term “photosynthesis” was introduced only a century later [5].

Photosynthesis plays a very important role in sustaining life. Plants are important sources of oxygen and organic matter for photosynthesis, and carbon dioxide is used to provide reactions. It is particularly important to maintain and preferably increase the positive effects of photosynthetic reactions to fully understand the process of photosynthesis and to identify any factors that may impair or enhance it [15].

This research explored Fe_3_O_4_ NPs, which can affect the morphological parameters of plants. Significantly, Fe_3_O_4_ NPs (25 nm, 24 mg/l, and 4 mg/l) extended the shoot length of common wheat. Other research has concluded that low concentrations of Fe_3_O_4_ NPs may have a dual effect on plant green mass. In research with tobacco (*Nicotiana tabacum*), Fe_3_O_4_ NPs (5 nm, 3 mg/l) directly reduced plant length and caused severe chlorosis or leaf bleaching, degrading chloroplasts with increasing chlorophyllase activity [22]. Research on sweet pepper (*Capsicum annuum*) indicates that Fe_3_O_4_ NPs (52.4 nm, 0.05 mmol/l) increase shoot length and the number of shoots [14]. Comparing these studies, it can be concluded that a significant factor is the size of NPs; very small NPs are able to more easily enter plant cells and migrate through plant vascular tissue, affecting leaf chloroplast cells [13].

Penetration of NPs into chloroplasts can result in both positive and negative effects on chloroplast functionality. NPs, when present in small amounts in a cell, can bind to one of the protein complexes in the electron transport chain and accelerate electron transport between photosystems. Fe_2+_ and Fe_3+_ ions are obtained from NPs in the cell, which could act as electron donors and acceptors in PSII and significantly accelerate and improve the quality of electron transport [19]. If NPs enter chloroplast cells in large quantities and form agglomerates, the chloroplast cell, its wall, and other structures such as granules may be mechanically damaged [22].

The transport of larger NPs through plant tissues is difficult, so they usually do not reach the leaf cells but remain in the roots, where the plant breaks them down into Fe_2+_ ions, which are then used to form porphyrin rings that are the basis for chlorophyll a and chlorophyll b synthesis. Improving the synthesis of the structures required for photosynthesis also increases the efficiency and intensity of photosynthesis, synthesises carbohydrates in the plant, and directly promotes leaf growth [19].

Fe_3_O_4_ NPs at concentrations of 1 and 4 mg/l significantly reduced all identified parameters important for photosynthesis. There was a statistically significant reduction in the light absorption of the samples at 645 and 663 nm compared to the control. Chlorophyll a, chlorophyll b, and total chlorophyll also decreased. NPs at a concentration of 2 mg/l significantly reduced both absorbances at 663 nm and the amount of chlorophyll a. Chlorophyll *b* and total chlorophyll were reduced insignificantly. Although Fe_3_O_4_ NPs (2.4 mg/l) increased shoot length by morphological measurements, brown spots were observed on the leaves and leaf tips, confirming chloroplast cell damage and possible chlorosis. In general, Fe_3_O_4_ NPs entered the seedlings of common wheat, as the experimental groups differed only in the presence or absence of NPs.

During our research, morphological parameters and parameters important for photosynthesis were also determined for common wheat seedlings grown in ZnO NPs. Other research has shown a dual effect of ZnO NPs, both positive and negative, on both morphological and photosynthetic parameters. This research showed only a positive effect, and this effect was observed at only two concentrations. A statistically significant increase in shoot length was observed for plants grown with 4 mg/l ZnO NPs. At the same time, chlorophyll a, chlorophyll b, and total chlorophyll insignificantly increased in plants at these concentrations, so it can be considered that ZnO (4 mg/l) did not affect photosynthetic parameters. In other studies, ZnO has been shown to increase the length of both seedlings and roots in a variety of plants. In addition, if the seedling leaves are extended, the total amount of chlorophyll is also positively affected [18]. However, this relationship was not observed in this study. There was also a significant increase in chlorophyll b, but this was increased in plants grown with a ZnO NP concentration of 1 mg/l instead of 4 mg/l. Seedling shoot length increased only with ZnO NPs at the highest available concentration and not in plants with 1 mg/l. Therefore, the lowest concentration of ZnO NPs (1 mg/l) better penetrated wheat seedlings. ZnO is likely to produce the Zn_2_^+^ microelements needed by plants, which directly increase chlorophyll content, improve seed germination, and promote early flowering [18]. Higher concentrations of ZnO NPs did not affect photosynthetic parameters because, as found in another study, higher concentrations of ZnO cause a decrease in the transport of substances in the vacuole. Zn compounds accumulate in the vacuole. Vacuole dysfunction occurs, which can lead to lower photosynthesis intensity and plant growth capacity [12]. This study used ZnO NPs with a size of 32 nm, which could also hinder both the penetration of NPs into wheat seedlings and their further movement through the vascular tissue, possibly due to the relatively small effect of ZnO on common wheat seedlings. However, ZnO NPs have the potential to be used to improve the intensity of photosynthesis.

In general, this research has shown that different NPs can affect the seedlings of common wheat in very different ways. A positive effect was observed on the length of the wheat leaves caused by both ZnO NPs and Fe_3_O_4_ NPs. However, photosynthesis was affected very differently by each type of NP. The photosynthetic intensity was reduced by Fe_3_O_4_ nanoparticles at 2 and 4 mg/l, but ZnO NPs at 1 mg/l increased the intensity. However, research on these NPs needs to be continued. The optimal size of NPs should be clarified so that they can penetrate plants as easily as possible. It may be useful to use NPs in research that are already present in the environment rather than those are artificially synthesised, as NPs that already exist in nature may be better adapted to entering the plant. Research should also be continued into the photosynthesis process itself, and it remains to be seen which stages of the photosynthetic reactions are affected by NPs. The interaction between photosynthesis and nanoparticles has the potential to be very positive, and there is an opportunity to improve the intensity of photosynthesis so that plants get more oxygen and absorb more carbon dioxide.

## 5. Conclusions

Based on the data obtained in this research, both NPs affected *Triticum aestivum* L. seedling growth by significantly (*P*<0.05) increasing the shoot length of the seedlings. Both NPs affected photosynthetic parameters with significant (*P*<0.05) potential to increase the intensity of photosynthesis or to degrade photosynthesis. The type and size of NPs are the main factors that create an impact. All concentrations, not only high but also low, can pose a significant threat to living organisms and to the environment, as well as improve it; therefore, the management of nature and the use of nanoparticles in manufacturing need to be improved. Future studies are needed to obtain knowledge about which stages of photosynthetic reactions are affected by NPs and how these particular NPs affect photosynthetic reactions.

## Figures and Tables

**Table 1:** Chlorophyll a and b and total chlorophyll content of samples of *Triticum aestivum* L. after exposure to Fe_3_O_4_ NPs.

Concentration of Fe_3_O_4_NPs (mg/l)	Amount of chlorophyll a (*μ*g/g) ± SD	Amount of chlorophyll b (*μ*g/g) ± SD	Amount of total chlorophyll (*μ*g/g) ± SD
Control	0.88 ± 0.18	0.35 ± 0.073	1.23 ± 0.25
1	0.46 ± 0.18*^∗^*	0.21 ± 0.064*^∗^*	0.67 ± 0.23*^∗^*
2	0.69 ± 0.21*^∗^*	0.32 ± 0.097	1.01 ± 0.30
4	0.64 ± 0.12*^∗^*	0.24 ± 0.056*^∗^*	0.88 ± 0.17^∗^

**Table 2:** Chlorophyll a and b and total chlorophyll content of samples of *Triticum aestivum* L. after exposure to ZnO NPs.

Concentration of ZnO NPs (mg/l)	Amount of chlorophyll a (*μ*g/g) ± SD	Amount of chlorophyll b (*μ*g/g) ± SD	Amount of total chlorophyll (*μ*g/g) ± SD
Control	0.51 ± 0.138	0.21 ± 0.065	0.71 ± 0.20
1	0.58 ± 0.088	0.26 ± 0.051*^∗^*	0.84 ± 0.12
2	0.53 ± 0.090	0.21 ± 0.030	0.73 ± 0.096
4	0.55 ± 0.071	0.24 ± 0.053	0.79 ± 0.082

## Data Availability

The data presented in this study are available on request from the corresponding author.
